# Receiver operating characteristic–derived thresholds for early validation of myocardial injury in a rat ischemia-reperfusion model

**DOI:** 10.3389/fphys.2026.1923294

**Published:** 2026-10-07

**Authors:** Rani Wainer-Shlomo, Michal Barsheshet Peer, Mona Shehadeh, Shaul Atar, Nir Qvit, Offir Ertracht

**Affiliations:** 1 Bar-Ilan University, Azrieli Faculty of Medicine, Safed, Israel; 2 Cardiac Rehabilitation, The Cardiovascular Department, Tzafon Medical Center, Poriya, Israel; 3 The Cardiovascular Research Laboratory, Galilee Medical Center, Nahariya, Israel; 4 Clinical Laboratories Division, Galilee Medical Center, Nahariya, Israel; 5 The Cardiology Department, Galilee Medical Center, Nahariya, Israel

**Keywords:** Cardiac troponin I, echocardiography, ischemia-reperfusion, myocardial infarction, reperfusion-injury

## Abstract

**Background:**

Myocardial infarction (MI) is a major manifestation of cardiovascular disease and a leading cause of morbidity and mortality worldwide. In experimental research, rat ischemia-reperfusion (IR) model is widely used to evaluate cardioprotective strategies for translational and clinical purposes. However, early confirmation of successful MI induction often relies on subjective intraoperative observations, and objective criteria for verifying myocardial injury early after IR remain poorly standardized, potentially limiting reproducibility and leading to unnecessary loss of time and resources. This study aimed to establish objective early indicators of myocardial injury following IR surgery in rats and to derive preliminary receiver operating characteristic (ROC)-based diagnostic thresholds for early confirmation of cardiac injury.

**Methods:**

Rats subjected to IR or sham surgery were evaluated during the first postoperative days using blood biochemistry and echocardiography. Based on functional, hemodynamic, and histological assessments performed at day 56, a subgroup of IR-operated animals with preserved cardiac function and minimal fibrosis was classified as non-injured and served as the reference group for subsequent ROC analyses.

**Results and discussion:**

Cardiac troponin I (cTnI) emerged as a reliable threshold marker for distinguishing animals with significant cardiac injury, exceeding > 12,000 ng/L within 24 h after injury. Lactate dehydrogenase (LDH) and aspartate aminotransferase (AST) also demonstrated reliable threshold values for identifying significantly injured animals (> 780 U/L, > 960 U/L, respectively). In parallel, echocardiography revealed threshold-based evidence of early systolic dysfunction in injured animals, with reduced fractional shortening (≤ 35%,( fractional area change (≤ 55%) and ejection fraction (≤ 70%), providing functional confirmation of impaired cardiac performance. Together, these ROC-derived thresholds provide a preliminary practical panel of biomarkers and echocardiographic parameters for early *in vivo* identification of myocardial injury following IR in rats, with the potential to improve experimental consistency and facilitate early model validation.

## Introduction

Cardiovascular disease is the leading global cause of morbidity and mortality, imposing a substantial economic burden (Virani et al., 2020). Myocardial infarction (MI) is a primary contributor to cardiovascular diseases-related deaths. MI results from coronary ischemia and reperfusion and may lead to irreversible cardiac damage. Ischemia-reperfusion (IR) injury induces complexed molecular and inflammatory processes that contribute to cardiomyocyte death and adverse ventricular remodeling, as superbly demonstrates in experimental IR models (Zuo et al., 2025). Due to the heart’s limited regenerative capacity, IR might cause permanent structural remodeling and fibrosis (Roger, 2013). Clinically, early reperfusion by thrombolytic therapy, balloon angioplasty, or primary percutaneous coronary intervention (PPCI) is the standard therapeutic approach, as it reduces mortality and preserves cardiac function (Michael et al., 1999).

Commonly experimental MI is induced by surgical ligation of the left anterior descending (LAD) coronary artery. This approach can be performed with or without subsequent reperfusion to create IR injury model or permanent ligation model, respectively (De Villiers and Riley, 2020; Lucchesi, 1990). In contemporary research, the rodent IR and permanent ligation models are the most widely used to study cardiac injury (De Villiers and Riley, 2020). The IR model is extensively used to investigate ischemic mechanisms and evaluate novel interventions, however, most studies focus on pre-conditioning strategies applied before injury (Song et al., 2023).

Nowadays, the IR model is more relevant to the clinical arena, as already the majority of patients with MI undergo reperfusion therapy (Yang et al., 2022). Future studies may research complementary post PCI treatments, or the efficacy of pharmaceuticals given at pre-reperfusion. Consequently, IR represents the most common clinical scenario, increasing the translational relevance of this model.

An inherent problem of IR surgeries is the lack of standardized methods and objective parameters, that indicate the presence and the extent of cardiac damage inflicted, as early as possible after the procedure. Previous studies have used heterogeneous approaches to confirm successful induction of myocardial ischemia. Importantly, methodological reporting remains inconsistent, particularly regarding the confirmation of successful MI induction. A systematic evaluation of experimental MI studies found substantial variability in the reporting of procedural validation, with 47% of studies not reporting confirmation of ischemia and 73% not reporting confirmation of reperfusion (Lindsey et al., 2021). When confirmation was performed, commonly used intraoperative indicators include electrocardiography changes as well as visual observations, such as myocardial color changes, arrhythmias, and abnormal wall motion (Jorge et al., 2011; Farag et al., 2023). Although these observations provide immediate feedback during surgery, visual indicators are inherently qualitative and operator-dependent. Furthermore, although electrocardiography provides a more objective real-time assessment of successful coronary occlusion, it primarily confirms the occurrence of ischemia rather than the extent of the resulting myocardial injury. Its acquisition and interpretation also require appropriate instrumentation and expertise. This limitation is particularly relevant in experimental IR research, where studies commonly evaluate cardioprotective interventions administered before the ischemic insult or around the onset of reperfusion, with the aim of attenuating subsequent myocardial injury (Wever et al., 2015; Song et al., 2023). Such interventions are specifically designed to modify the myocardial response to IR and may substantially influence infarct size and other indices of myocardial damage. Consequently, post-IR measurements may reflect both variability in the initial ischemic injury and the cardioprotective effect of the intervention itself, complicating the objective confirmation of comparable initial myocardial injury across experimental groups. Importantly, despite undergoing an apparently identical IR protocol, the extent of myocardial injury may vary substantially among animals. This variability may reflect anatomical differences in coronary artery branching patterns as well as technical factors, particularly the precise location of coronary artery ligation, both of which have been shown to influence infarct size in rat models (Kainuma et al., 2017). Consequently, some animals may exhibit minimal persistent functional or structural injury despite undergoing the complete IR procedure.

Previous studies have used circulating cTnI and echocardiographic parameters to assess myocardial injury and cardiac dysfunction in rodent MI models (Frobert et al., 2015; Daud et al., 2021). However, objective early cutoff values for distinguishing injured from minimally injured animals following IR remain insufficiently defined.

Studies evaluating interventions initiated only after completion of the IR insult and initial injury establishment are less represented in the preclinical literature (Song et al., 2023). In such post-injury designs, objective confirmation of myocardial injury before treatment initiation is important for establishing a comparable baseline injury. These considerations emphasize the need for standardized, non-terminal, and objective criteria for early confirmation of myocardial injury after IR, thereby improving reproducibility and comparability across studies.

The early evaluation of the surgery effect is of paramount importance, as it may improve experiment methodology. Early evaluation of procedural success is important for improving methodological rigor. Establishing objective thresholds for selected predictor variables may improve reproducibility, prevent the unnecessary continuation of experiments in animals with insufficient injury, and conserve time and resources.

We hypothesize that analyzing circulating and echocardiography would allow the establishment of objective threshold values for accurately defining cardiac damage. Herein, we define objective indicators for confirming early cardiac damage following IR surgery in rats and establish preliminary ROC-derived diagnostic thresholds for model validation. These proposed criteria may facilitate early *in vivo* identification of cardiac damage, improve experimental consistency, and provide a practical framework for future validation studies.

## Materials and methods

All animal experiments were approved by the institutional animal ethics committee of Bar-Ilan University, Israel (protocol number BIU-MD-IL-2211-16-4), and were conducted in accordance with the Guide for the Care and Use of Laboratory Animals (US National Research Council, Eighth Edition, 2011). All procedures complied with institutional and national guidelines for the care and use of laboratory animals. This study was reported in accordance with the ARRIVE 2.0 guidelines.

Male Sprague-Dawley rats (Envigo Ltd., Jerusalem, Israel) were housed under controlled temperature and humidity on a 12:12 h light/dark cycle, with free access to standard rodent chow and water.

### Study design

The study included 60 rats that underwent baseline (BL) assessments, including blood sampling and echocardiography. The animals were then randomly allocated to either an IR surgery group (n=51) or a sham-operated group (n=9). Subsequentially, all animals underwent blood chemistry and echocardiography at 1 and 3 days post procedure, respectively. Animals were sacrificed on day 56 to allow the possible development of complete cardiac remodeling post-surgery. Based on the final histological and physiological assessments, animals exhibiting minimal cardiac injury were classified as non-injured. Subsequent comparisons were conducted between sham-operated animals (n=9), non-injured animals (n=10), and injured animals (n=41). The criteria used to define injured animals are detailed in subsection *Final diagnosis and classification of myocardial injury*.

**Figure 1** presents the study timeline.

**Figure 1:**
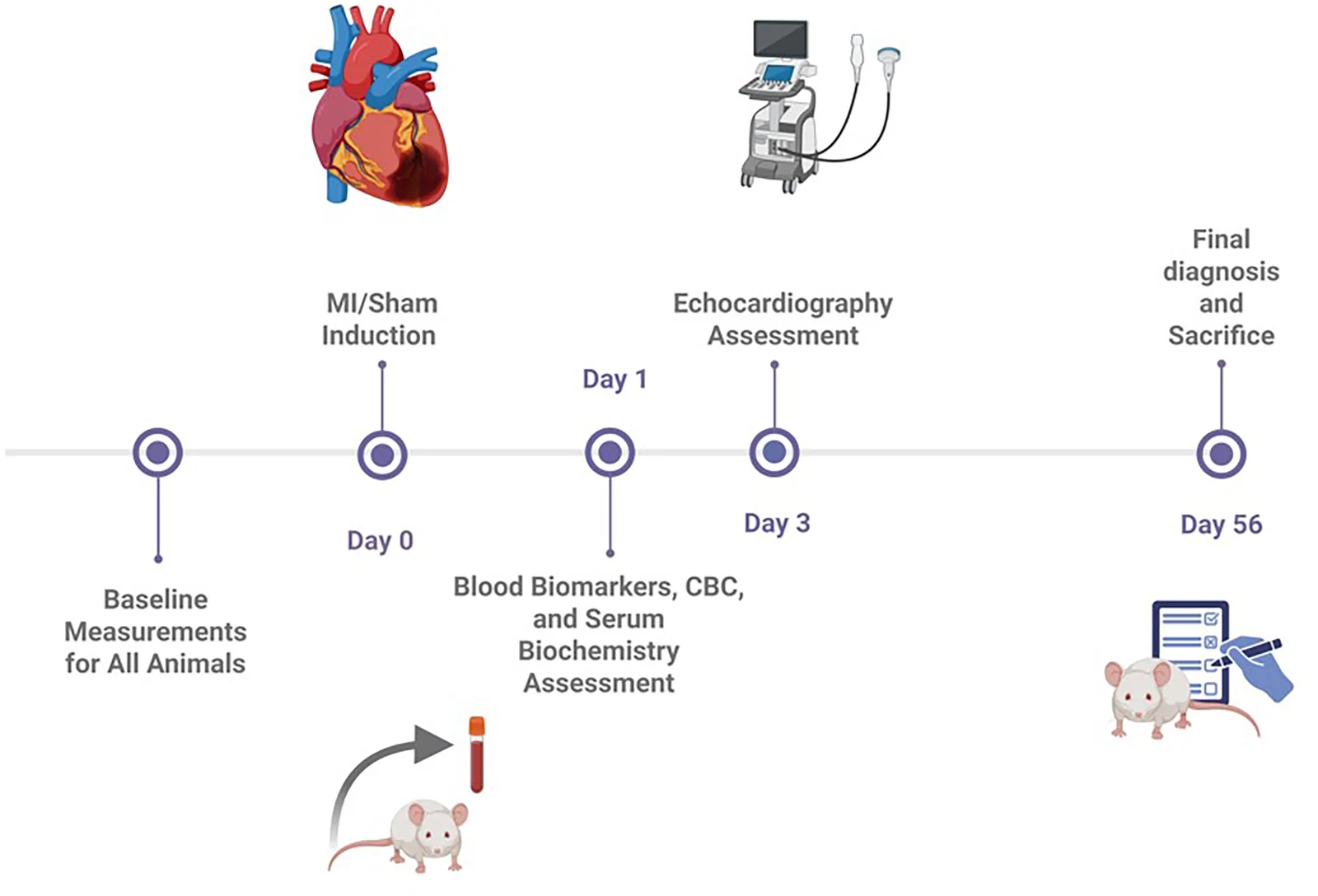
Study timeline. Schematic overview of the experimental protocol. The study spans over 56 days and includes baseline (BL) assessment, IR induction, and longitudinal functional and molecular evaluations. BL measurements were performed prior to IR surgery. Hematological and echocardiographic assessment were conducted a day and 3 days post-surgery, respectively. The protocol lasted 56-day to allow complete cardiac remodeling.

### Blood analysis

To allow recovery and achieve the change in relevant biochemical parameters, blood samples were obtained only 24 h post-surgery. Blood was collected under isoflurane anesthesia from the tail vein. Complete blood count (CBC) was measured using the Sysmex XN-1000™ Hematology Analyzer (Sysmex America Inc., Lincolnshire, IL, USA). Parameters included indices related to oxygen-carrying capacity and erythrocyte characteristics as red blood cell count (RBC), mean corpuscular hemoglobin concentration (MCHC), and mean corpuscular volume (MCV). Additionally, total blood protein levels, leukocyte counts (white blood cells (WBC)) and markers of systemic inflammation, including the neutrophil-to-lymphocyte ratio (NLR).

Serum biochemistry was obtained after centrifugation of whole blood (4 °C, 2,500 × g, 10 min) and analyzed on Alinity systems (Abbott Core Laboratory Systems, Lake Forest, IL, USA). Cardiac injury markers included among other creatine kinase (CK), cardiac troponin I (cTnI), aspartate aminotransferase (AST), and lactate dehydrogenase (LDH).

### Echocardiography

To minimize the confounding effects of post-operative local edema on cardiac function assessment, echocardiography was performed on day three post-surgery. Rats were lightly sedated with intramuscular ketamine (29 mg/kg) and xylazine (4.3 mg/kg) (Liu and Rigel, 2009). Echocardiography was performed using a Vivid *i* scanner (GE, Haifa, Israel) equipped with a 10S phased-array pediatric transducer (12 MHz; depth 2.5 cm; ≥315 frames/s). Two parasternal short-axis views-apical myocardial IR injury and papillary muscle (PM)-were obtained. We evaluated both structural parameters and functional parameters. The structural parameters included: LV cross-sectional areas at systole (LVESA) and diastole (LVEDA), LV internal diameters at systole (LVIDs) and diastole (LVIDd), septal and free wall thicknesses. The functional parameters included fractional shortening (FS), fractional area change (FAC), and ejection fraction (EF). FS was calculated from LVIDd and LVIDs, and FAC from LVEDA and LVESA. Left ventricular end-diastolic and end-systolic volumes were estimated from M-mode-derived LVIDd and LVIDs, respectively, using the Teichholz method (Teichholz et al., 1976), and EF was subsequently calculated from the estimated ventricular volumes. Given the regional nature of myocardial injury following LAD occlusion, EF was included as a complementary measure of global systolic function, as FS is based on one-dimensional measurements and FAC may be influenced by endocardial border tracing. Post-injury echocardiographic analysis was performed using short-axis measurements at the apical level, as myocardial dysfunction following infarction is most prominently expressed in this view, allowing clearer visualization of the extent of regional injury.

### Ischemia-reperfusion model

LAD coronary artery ligation was performed as described previously (Bhindi et al., 2006; Ertracht et al., 2011). Briefly, rats were anesthetized with intramuscular ketamine (87 mg/kg) and xylazine (13 mg/kg), intubated, and mechanically ventilated (80–90 breaths/min; 1–2 mL/100 g tidal volume). A left thoracotomy (third intercostal space) was performed, the pericardium opened, and the LAD identified. A 5/0 Prolene thread equipped with curved cutting needle was used to perform a stitch that was placed through the myocardium at a slightly greater depth than the perceived level of the LAD artery. A reversible ligature was applied to occlude the LAD for 30 min, followed by its rapid release to restore flow. Sham-operated animals underwent a similar procedure except for ligation of the LAD coronary artery. Finally, the chest was closed, skin sutured, analgesics administered, and animals allowed to recover.

### Final diagnosis and classification of myocardial injury

Final classification of IR-injured or non-injured animals was based on comprehensive cardiac assessment performed at the end of the experimental protocol (day 56). This multi-modal evaluation included pressure-volume loop analysis and histological examination, as previously described (Pacher et al., 2008). Animals were classified as non-injured when cardiac function was preserved, defined as EF > 70%, stroke volume > 180 µL, and cardiac output > 45,000 µL/min, together with minimal histological evidence of fibrosis (≤ 2 mm²) (data not shown).

Animals classified as IR-injured exhibited impaired cardiac function and histological evidence of myocardial injury. This final multi-modal classification served as the reference standard for all receiver operating characteristic (ROC) analyses. Echocardiographic measurements and histological quantification were not performed blinded to group allocation.

### Statistical analysis

Data are expressed as mean ± SD. Statistical analysis was performed using SigmaStat for Windows v3.11 (Systat Software Inc., San Jose, CA, USA). Normality of data distribution was assessed using the Shapiro–Wilk test, and homogeneity of variance was evaluated using Brown-Forsythe test prior to parametric comparisons. All analyzed variables met the assumptions of normality and homogeneity of variance; therefore, parametric tests were applied as described below. Comparisons among the three study groups were performed using a one-way Analysis of Variance (ANOVA), followed by Tukey’s *post hoc* test for multiple comparisons. A P value < 0.05 was considered statistically significant. Subsequently, differences between the non-injured and injured groups were assessed using an unpaired two tailed *t*-test, and only parameters that demonstrated P < 0.05 were included in the ROC analysis. ROC analysis was conducted to evaluate the ability of selected parameters to discriminate between non-injured and injured animals. ROC curves provide a graphical assessment of diagnostic performance by illustrating the trade-off between sensitivity and specificity across a range of thresholds.

For each parameter, sensitivity and specificity were calculated across all possible threshold values, and the area under the ROC curve (AUC) was determined as a measure of diagnostic performance. The optimal cutoff value was defined using the Youden index (J-score), calculated as sensitivity plus specificity minus one. The threshold corresponding to the maximal J-score was selected as the optimal cutoff for distinguishing between groups. In cases where multiple thresholds yielded identical J-score values, the cutoff with the highest specificity was selected. This conservative approach was implemented to maximize the certainty that animals classified as “injured” truly exhibited myocardial injury, thereby minimizing false-positive classification. Although this strategy may reduce sensitivity and increase the likelihood of false-negative cases, it prioritizes diagnostic specificity and ensures that downstream analyses are conducted on a highly reliable injured cohort.

## Results

### Baseline measurements

BL physiological, hematological, and echocardiography functional parameters were assessed to confirm the health status of the animals prior to any intervention. Body weight (BW), CBC, serum biochemistry and echocardiographic indices are summarized in **Tables 1** and **2**.

**Table 1:** BL hematology and serum biochemistry profiles.

Parameter	Mean ± SD (n=60)
Alanine transaminase (ALT) [U/L]	63.4 ± 17.0
Aspartate aminotransferase (AST) [U/L]	76.2 ± 17.7
Alkaline phosphatase (AlkP) [U/L]	302.6 ± 56.4
Calcium [mg/dL]	10.2 ± 0.7
Creatine kinase (CK) [U/L]	235.1 ± 88.8
Chloride [mM]	98.8 ± 4.7
Creatinine [mg/dL]	0.32 ± 0.03
Glucose [mg/dL]	191.1 ± 62.7
Potassium [mM]	4.2 ± 0.6
Lactate dehydrogenase (LDH) [U/L]	195.1 ± 94.6
Magnesium [mg/dL]	1.8 ± 0.2
Sodium (Na) [mM]	140.2 ± 5.5
Phosphorus [mg/dL]	6.9 ± 0.9
Triglyceride [mg/dL]	75.0 ± 17.6
Urea [mg/dL]	15.6 ± 1.8
Cardiac troponin I (cTnI) [ng/L]	< LOD
White blood cell count [10^3/ul]	9.7 ± 2.1
Red blood cell count [10^6/ul]	6.2 ± 1.0
Hemoglobin [g/dl]	12.6 ± 2.2
Platelets [10^3/ul]	534.6 ± 330.4
Hematocrit [%]	38.2 ± 5.7
Neutrophils [%]	7.1 ± 1.8
Lymphocytes [%]	89.6 ± 2.5
Neutrophil-to-lymphocyte ratio (NLR)	0.08 ± 0.02
Platelet-to-lymphocyte ratio (PLR)	7.0 ± 1.4
Monocytes [%]	2.7 ± 1.7
Eosinophils [%]	0.7 ± 0.2

The table summarizes BL blood measurements. n=60. Data are presented as mean ± SD. Baseline cTnI concentrations were below the assay limit of detection (LOD) and are therefore reported as < LOD rather than mean ± SD.

**Table 2:** BL echocardiographic profiles.

Level and phase Parameter	Apex level systole	Apex level diastole
Left ventricular internal diameter (LVID) [mm]	3.11 ± 0.65	6.19 ± 0.86
Septum width (Interventricular septum (IVS) [mm]	3.09 ±0.53	2.08 ± 0.55
Free wall width (Left ventricular posterior wall (LVPW) [mm]	2.55 ± 0.32	1.60 ± 0.23
Fractional shortening (FS) [%]	50 ± 6	
Fractional area change (FAC) [%]	74 ± 6	
Ejection fraction (EF) [%]	86 ± 5	

The table summarizes BL physiological echocardiographic measurements. n=60. Data are presented as mean ± SD.

Specifically, mean rat’s BW was 258 ± 45 g., key CBC findings included: White blood cells (WBC) count 9.6 ± 2.1 × 10³/μL, red blood cell count (RBC) count 6.3 ± 1.1 × 10^6^/μL, platelet count 508.5 ± 320.2 × 10³/μL, neutrophils 7.0 ± 1.8%, and lymphocytes 89.6 ± 2.5%. All within normal ranges for healthy young rats.

In the BL serum chemistry, we measured calcium 10.3 ± 0.5 mg/dL, creatine kinase (CK) 228.8 ± 90.2 U/L, glucose 195.8 ± 63.8 mg/dL and triglycerides 76.2 ± 16.4 mg/dL-consistent with normal young rat physiology (**Table 1**). Baseline cTnI concentrations were below the assay limit of detection (<10 ng/L).

Echocardiographic assessment (**Table 2**) demonstrated that at the AP level the mean FS was 50 ± 6%, FAC 74 ± 6%, and EF 86 ± 5%, indicating normal systolic function.

Overall, the rats demonstrated BWs, hematological profiles, serum biochemistry, and cardiac function consistent with healthy young rats.

Final classification of animals as IR-injured or non-injured animals was determined at day 56 using a multi-modal cardiac assessment integrating echocardiographic, hemodynamic, and histological criteria, which served as the reference standard for all downstream analyses. This injury-based classification is further illustrated in **Figure 2**.

**Figure 2:**
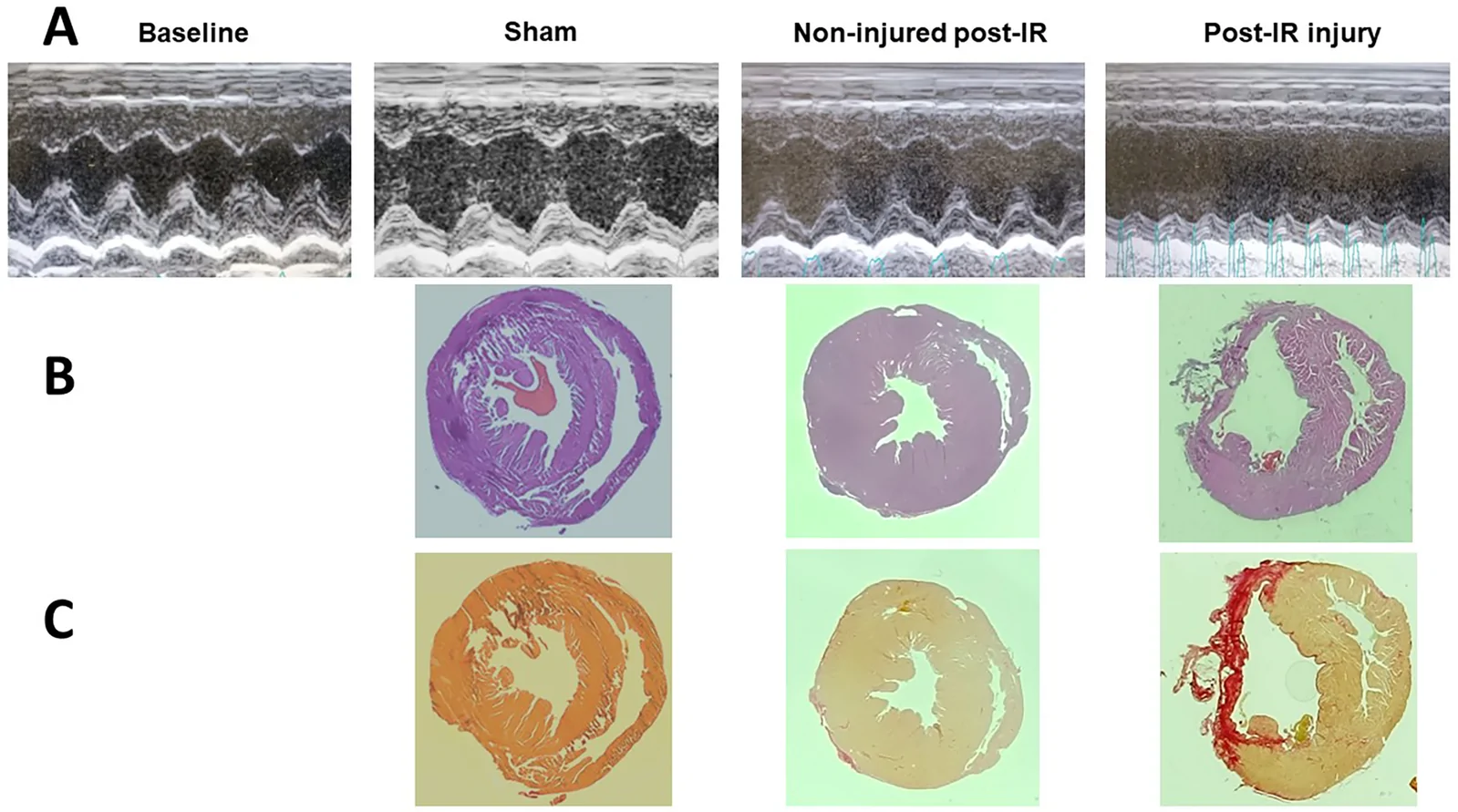
Representative confirmation of damage post IR. **(A)** M-mode echocardiographic images at baseline, and 3 days following sham or IR surgery with/without functional and histological cardiac damage. **(B)** Representative histological hematoxylin and eosin (H&E) staining sections of sham, IR-injured and non-injured hearts at day 56. **(C)** Representative histochemical picrosirius red staining sections of sham, IR-injured and non-injured hearts at day 56. Picrosirius red highlights collagen deposition (fibrosis) in red, indicating structural remodeling following myocardial injury.

### Post operation measurements

Blood and echocardiography parameters are summarized in **Table 3**. A noticeable level of cTnI was found in all groups, 960.2 ± 537.9 ng/L, 6063 ± 3349 ng/L, and 43,722 ± 21,74 ng/L in the sham, non-injured and injured groups, respectively. While sham group’s cTnI was lower yet comparable to the non-injured group’s (P>0.05), significant differences were found between each group to the injured one (P<0.0001 *vs.* sham and P<0.001 *vs.* non-injured groups, **Table 3**). Similarly, LDH was 490 ± 211.6 U/L and 423.7 ± 185.5 U/L in the sham and non-injured groups (P>0.05), and 1,216.0± 672.8 U/L in the injured group (P<0.05 *vs.* both groups, **Table 3**). AST and CK were comparable among the experimental groups (P>0.05, **Table 3**).

**Table 3:** Hematological and echocardiographic selected parameters following IR-induced myocardial injury.

Parameter	Sham (n=9)	Non-injured (n = 10)	Injured post-IR (n = 41)	Statistical significance
Cardiac troponin I (cTnI) [ng/L]	960.2 ± 537.9	6,063 ± 3,349	43,722 ± 21,743 ^****,###^	*P* < 0.0001
Aspartate aminotransferase (AST) [U/L]	855.2 ± 463.5	726.4 ± 407.1	1,098.0 ± 405.9	*NS*
Lactate dehydrogenase (LDH) [U/L]	490.5 ± 211.6	423.7 ± 185.5	1,216.0 ± 672.8 ^*,##^	*P* < 0.01
Creatine kinase (CK) [U/L]	302.9 ± 124.1	211.1 ± 108.3	421.2 ± 319.0	*NS*
Fractional shortening (FS) [%]	39.8 ± 3.5	39.4 ± 2.8	24.9 ± 4.5 ^****,####^	*P* < 0.0001
Fractional area change (FAC) [%]	61.5 ± 4.8	66.7 ± 8.8	45.2 ± 12.0 ^**,####^	*P* < 0.0001
Ejection fraction (EF) [%]	75.8 ± 4.2	75.6 ± 3.2	55.1 ± 7.2 ^****,####^	*P* < 0.0001

This table summarizes hematological and echocardiographic indices following the surgery. Data are presented as mean ± SD. Statistical analysis was performed using one-way ANOVA followed by Tukey’s *post hoc* test. *P < 0.05 vs. sham, **P < 0.01 vs. sham, ***P < 0.001 vs. sham, ****P < 0.0001 vs. sham, #P < 0.05 vs. non-injured, ##P < 0.01 vs. non-injured, ###P < 0.001 vs. non-injured, ####P < 0.0001 vs. non-injured.

All echocardiography functional parameters (FS, FAC and EF), were comparable between the sham and non-injured group, for example EF was 75.8 ± 4.2%, and 75.6 ± 3.2% in the sham and non-injured groups, respectively (P>0.05, **Table 3**). On the other hand, the injured groups’ echocardiography functional parameters were significantly attenuated in comparison to the other groups, e.g. mean injured EF was 55.1 ± 7.2% (P<0.001 *vs.* both groups, **Table 3**).

Collectively, these data indicate that sham and non-injured rats are comparable in all parameters, and cTnI, LDH, and the functional echocardiography parameters may serve as tools to separate between sham or non-injured to injured animals.

One of the research’s motivations is to find a way to distinguish between non-injured to injured animals. Accordingly, we specifically compared non-injured to injured by un-paired two tailed *t*-test. **Figure 3** summarizes the evaluated parameters. As can be seen except for CK and NLR, all parameters are highly significant between the groups. Specifically, in the injured group cTnI increases by ~seven times, AST by ~1.5 times, LDH by ~three times and PLR by ~1.3 times in comparison to the non-injured group (**Figure 3**).

**Figure 3:**
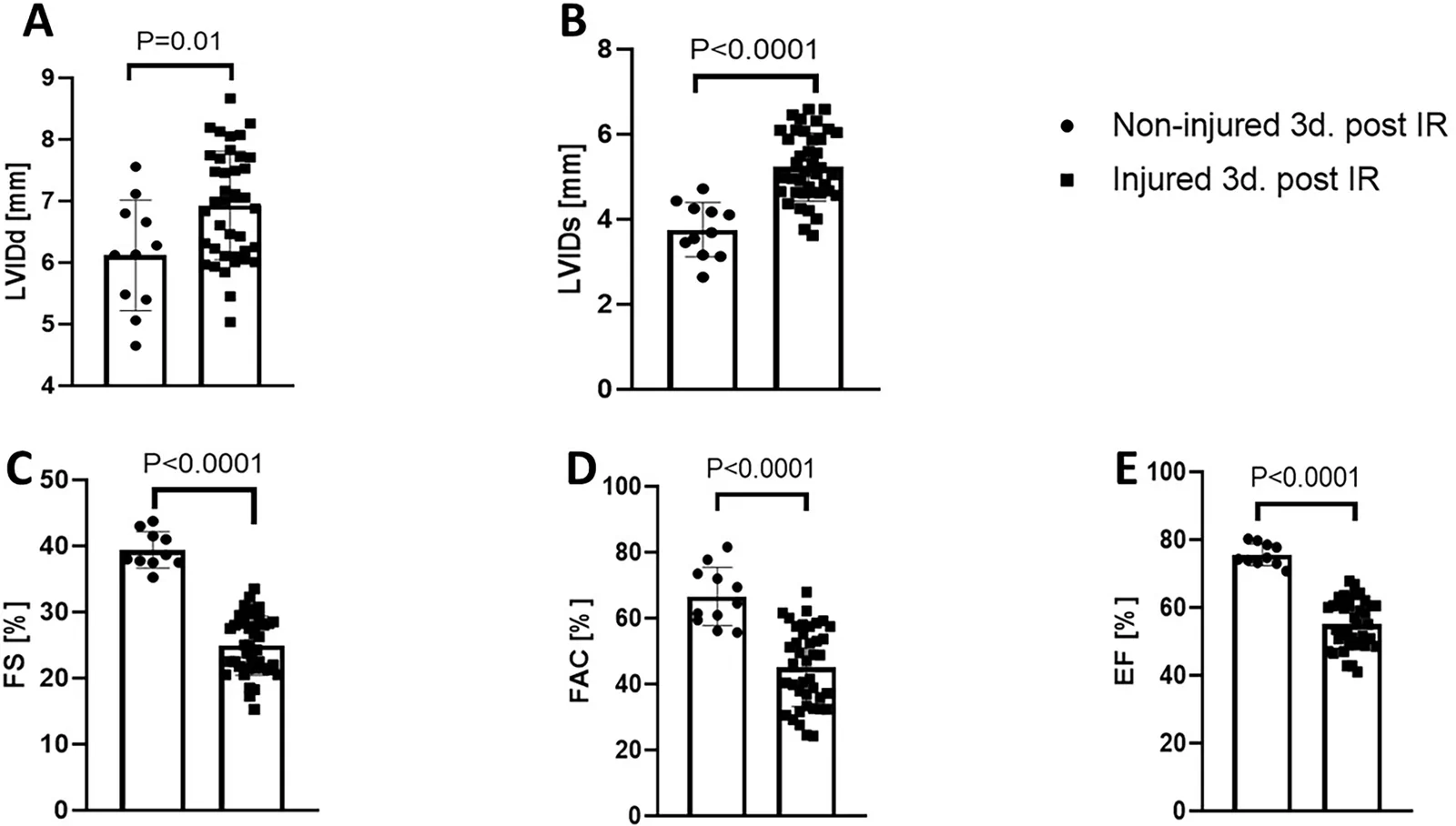
AP echocardiography indicators of cardiac structure and function. **(A)** Left ventricular internal diameter in diastole (LVIDd); **(B)** left ventricular internal diameter in systole (LVIDs); **(C)** fractional shortening (FS); **(D)** fractional area change (FAC); and **(E)** ejection fraction (EF). Values are presented as mean ± SD. Statistical analysis was performed using *t*-test. n=10 of non-injured rats, and n=41 of injured rats.

Further, both structural and functional echocardiography parameters, as soon as three days post IR were attenuated in the injured group in comparison to non-injured group. Particularly, LVIDd, and LVIDs increased by ~17% and 6%, respectively (P<0.01, **Figure 4**), and the functional parameters decreased by ~37.5%, ~32.3% and ~27% for the FS, FAC and EF, respectively (P<0.001, **Figure 4**).

**Figure 4:**
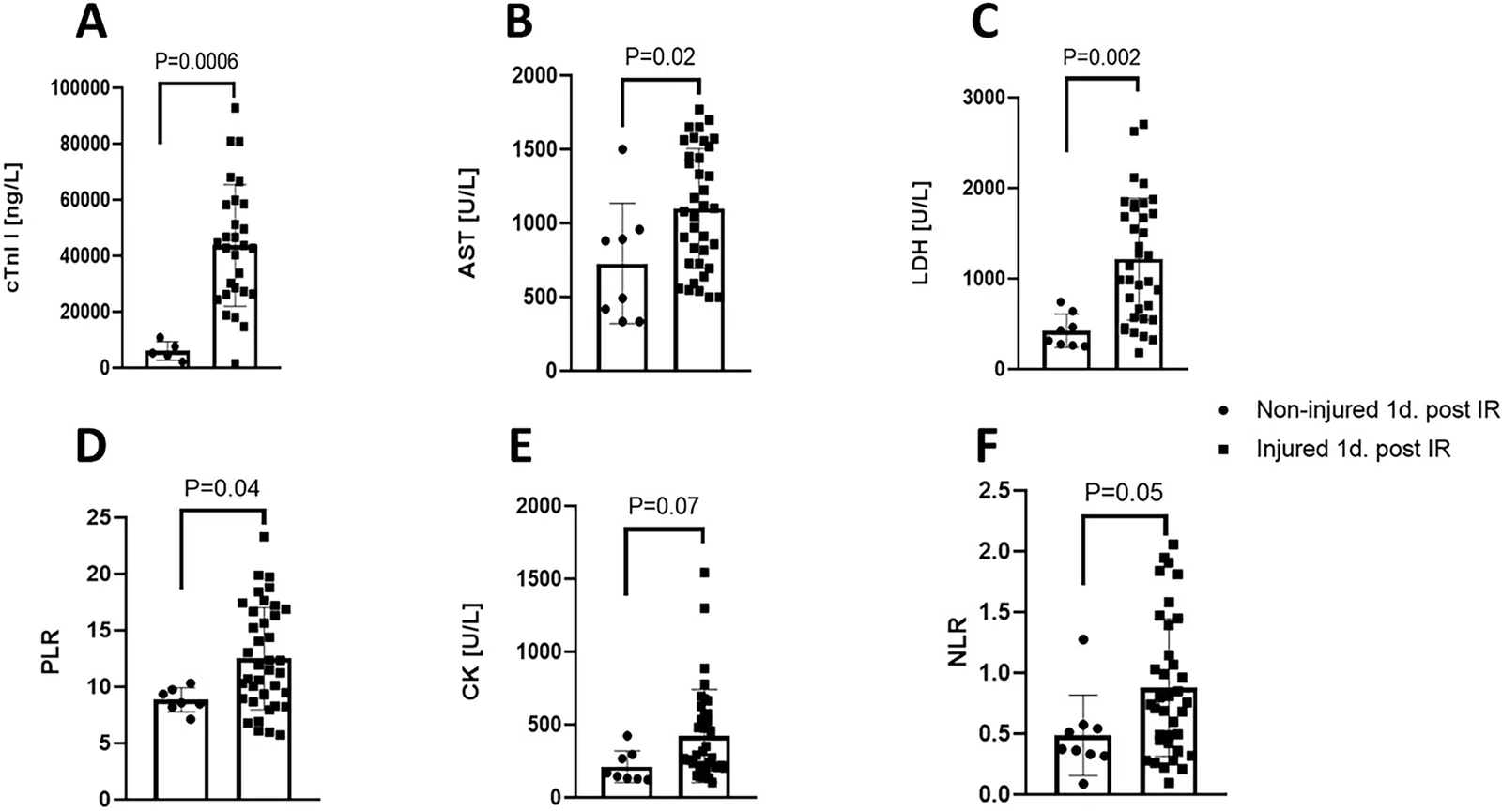
Circulating biomarkers of tissue injury. **(A)** Cardiac troponin I (cTnI); **(B)** aspartate aminotransferase (AST); **(C)** lactate dehydrogenase (LDH); **(D)** platelet-to-lymphocyte ratio (PLR); **(E)** creatine kinase (CK); and **(F)** neutrophil-to-lymphocyte ratio (NLR). Values are presented as mean ± SD. Statistical analysis was performed using *t*-test. n=10 of non-injured rats, and n=41 of injured rats.

To quantify the diagnostic accuracy of the significant differences between non-injured and injured animals we subjected them to ROC curve analysis. (**Table 4**; **Figure 5**). Although PLR, LVIDd, and LVIDs demonstrated significant differences between the non-injured and injured groups, considerable variability and overlap across groups, particularly with sham-operated animals, limited their diagnostic performance. Accordingly, these parameters yielded relatively low AUC values and were therefore excluded from further ROC-based threshold determination and subsequent analyses.

**Table 4:** Diagnostic performance and optimal cutoff values derived from receiver operating characteristic (ROC) analysis.

Parameter	AUC (95% CI)	Optimal cutoff	Sensitivity (%)	Specificity (%)
Hematological parameters				
Cardiac troponin I (cTnI) [ng/L]	0.97 (0.91- 1.00)	> 12,797	96.4	100.0
Lactate dehydrogenase (LDH) [U/L]	0.87 (0.76-0.97)	> 784.8	68.6	100.0
Aspartate aminotransferase (AST) [U/L]	0.76 (0.57-0.96)	> 963.4	58.3	87.5
Echocardiographic parameters				
Ejection fraction (EF) [%]	1.00 (1.00-1.00)	< 69.25	100.0	100.0
Fractional shortening (FS) [%]	1.00 (1.00-1.00)	< 34.38	100.0	100.0
Fractional area change (FAC) [%]	0.93 (0.86-1.00)	< 55.42	75.0	100.0

This table summarizes the diagnostic accuracy of circulating and echocardiographic parameters that differ between non-injured and injured groups. AUC values closer to 1 indicate higher diagnostic accuracy.

**Figure 5:**
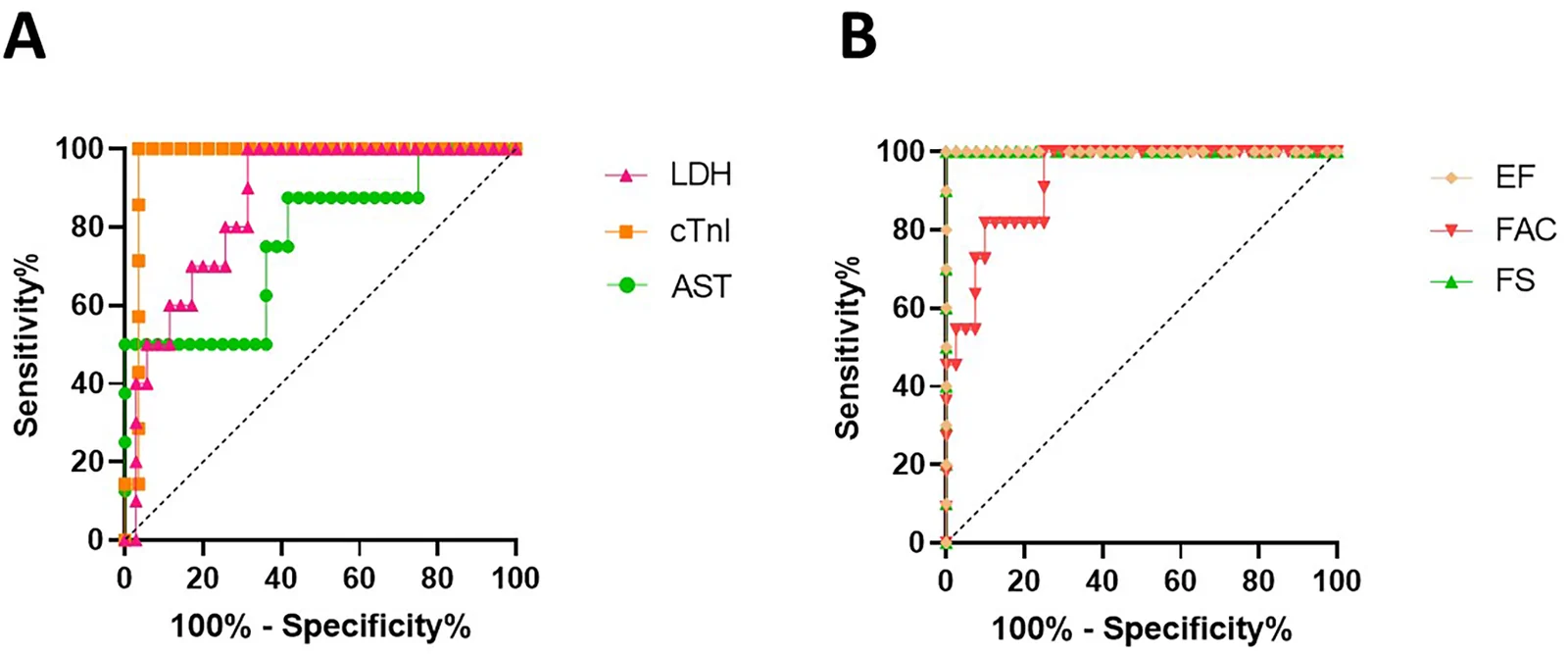
ROC analysis of **(A)** circulating and **(B)** echocardiographic indicators of cardiac damage.

ROC analysis defined the objective cutoff values for key circulating biomarkers and echocardiographic parameters associated with post IR cardiac damage. As shown in **Figure 5A** and **Table 4**, cTnI demonstrated the highest AUC among circulating parameters. Likewise, among echocardiographic indices (**Figure 5B**; **Table 4**), FS and EF showed the highest AUC values, indicating superior diagnostic performance compared to FAC. Although EF and FS demonstrated an AUC of 1.00 in the present cohort, this perfect separation should be interpreted cautiously, as the retrospective group classification may have contributed to inflation of the observed diagnostic performance.

For EF and FS, when multiple thresholds yielded identical J-score values, the cutoff with the highest specificity was selected. This conservative approach was implemented to maximize the certainty that animals classified as “injured” truly exhibited myocardial injury, thereby minimizing false-positive classification. Although this strategy may reduce sensitivity and increase the likelihood of false-negative cases, it prioritizes diagnostic specificity and ensures that downstream analyses are conducted on a highly reliable injured cohort.

## Discussion

The aim of this study was to establish standardized parameters for the early evaluation of IR-induced cardiac damage in rats, and to provide a practical guide for researchers for using them. Current approaches largely rely on subjective intraoperative assessments, and the lack of standardized early evaluation criteria limits the validity of IR model studies. To address this, we systematically evaluated blood biomarkers (e.g., cTnI, LDH) and echocardiographic measures (e.g. FS, FAC and EF), to identify reliable indicators of cardiac damage. We hypothesized that analyzing such parameters would establish objective thresholds for accurately defining cardiac damage. Importantly, ROC analyses were performed using a final multi-modal diagnosis integrating echocardiographic, invasive cardiac hemodynamic and histological findings as the reference standard, thereby reducing the risk of misclassification at defining non-injured *vs.* injured animals. To facilitate practical implementation across laboratories, the proposed cutoff values are presented as rounded, pragmatic thresholds rather than strictly adhering to the exact numerical values derived from ROC analysis. Although ROC analysis identifies mathematically optimal cutoff values, rounding these thresholds to clinically intuitive values often has a negligible effect on diagnostic accuracy while improving their practicality and ease of implementation. Importantly, application of these rounded thresholds to the present dataset did not alter their diagnostic sensitivity or specificity compared with the exact ROC-derived cutoff values. Therefore, pragmatic thresholds were selected to facilitate implementation and reproducibility in experimental laboratories. A similar pragmatic strategy has been reported in previous ROC-based diagnostic studies, where investigators selected clinically or experimentally practical thresholds close to the statistical optimum (Pepe, 2003; Ibis et al., 2025).

Interestingly, sham and non-injured animals exhibited normal histological features, despite demonstrating early (1 day post-insult) blood values that were markedly elevated compared with BL measurements. Similar observations have been reported previously, where sham surgery resulted in mild alterations in cardiac function despite the absence of substantial scar formation on histological evaluation (Nordbeck et al., 2013; Neckář et al., 2019; Bachner-Hinenzon et al., 2012). Furthermore, although infarct size can be quantified using triphenyl tetrazolium chloride or cardiac magnetic resonance imaging, these approaches are not suitable or practical for early validation in studies where animals are intended to undergo subsequent interventions. Therefore, the present work aimed to establish practical *in vivo* indicators that allow early confirmation of cardiac damage without terminating the experiment.

### Hematological indicators for early validation of IR-induced MI

Early enzymatic markers for suspected acute MI, such as AST, LDH, and CK, were historically used in clinical practice (Danese and Montagnana, 2016). Nowadays, cTnI is the gold standard for detecting myocardial necrosis (Canty Jr, 2022; Danese and Montagnana, 2016). Consistent with these observations, cTnI in the present study showed the most pronounced increase following IR procedure and demonstrated the highest sensitivity and specificity among the evaluated markers. In experimental MI models, circulating cTnI is a highly specific marker of cardiomyocyte injury and correlates with the extent of cardiac damage and left ventricular systolic dysfunction. In rat models, early cTnI elevation, together with echocardiographic parameters, has been shown to predict infarct severity and size (Frobert et al., 2015). In clinical settings, the kinetics of cTnI release are well characterized, with circulating levels typically rising within hours after ischemic injury, peaking around 24 h, and gradually declining over the following days (Babuin and Jaffe, 2005; Canty Jr, 2022). Comparable temporal patterns have been reported in rodents MI models, where biomarker elevation parallels the development of ventricular dysfunction and structural remodeling (Trueblood et al., 2005; Daud et al., 2021). Based on these kinetics and the diagnostic performance observed in our cohort, we propose a cTnI threshold of > 12,000 ng/L, LDH > 780 U/L, and AST > 960 U/L, at 24 h post-IR, as a practical criteria for identifying the substantially injured animals.

### Echocardiographic indicators for early validation of IR-induced MI

Functional echocardiography indices, including FS, FAC, and EF, may accurately reflect myocardial functional impairment than structural parameters (Jan and Tajik, 2017; Daud et al., 2021). We recommend thresholds for independent functional assessment at three days post-IR are FS ≤ 35%, FAC ≤ 55%, and EF ≤ 70%. Among these parameters, indices derived from M-mode measurements (FS and EF) appear to offer superior diagnostic performance for detecting cardiac damage, as indicated by their higher sensitivity, specificity, and AUC values compared with FAC. Nevertheless, conventional echocardiographic parameters have inherent limitations in the assessment of regional myocardial dysfunction. In particular, M-mode-derived measurements such as FS and EF rely on single-plane linear LV dimensions and geometric assumptions and may not fully reflect regional wall-motion abnormalities, particularly following MI, while area-based measurements such as FAC depend on accurate delineation of the endocardial border (Zacchigna et al., 2021). Notably, certain echocardiographic parameters, including EF and FS, demonstrated an AUC of 1.00, indicating perfect discrimination between non-injured and injured animals in the present dataset. While this finding likely reflects the marked functional separation between groups at this early post-injury time point, it should be interpreted cautiously given the relatively small sample size and requires validation in independent cohorts.

Importantly, ΔEF or percent change from BL EF may be more accurate than absolute echocardiographic parameters as those may be influenced by factors including acquisition settings, and operator experience. However, since BL echocardiographic measurements are not consistently performed in most experimental MI studies, we considered them less usable.

Consequently, absolute functional values remain the most practical and widely applicable approach in experimental settings. Importantly, the values obtained in the present study are consistent with those reported in previous IR studies (Li et al., 2025; Daud et al., 2021; Ertracht et al., 2014), suggesting that these absolute thresholds are reproducible across similar experimental conditions. Therefore, the proposed absolute values may serve as reliable and practical reference thresholds for early assessment of MI in comparable experimental frameworks.

### Functional similarity between sham and non-injured animals

An important finding of the present study was the similarity in functional cardiac parameters between sham-operated animals and animals subjected to IR surgery that were ultimately classified as non-injured. Although cTnI levels found, by *t*-test, to be significantly higher in the non-injured group (P<0.05), no differences were observed in other biochemical or echocardiography parameters. These findings suggest that the elevated troponin levels likely reflect cardiac damage that ultimately was not reflected with detectable functional or histological impairment. These findings indicate that non-injured animals may resemble sham-operated animals with respect to selected functional and structural cardiac outcomes. However, because these animals underwent actual myocardial ischemia and reperfusion, they cannot be considered equivalent to sham-operated controls, particularly with respect to molecular, inflammatory, or other biological responses not assessed in the present study. Accordingly, the present findings should not be interpreted as supporting the general replacement of sham-operated controls by non-injured animals.

## Conclusions

The aim of this study was to establish standardized criteria for early validation of successful cardiac damage induction in the rat IR model. Rather than identifying clinical biomarkers of MI, our goal was to define practical indicators that allow investigators to confirm model induction during the early post-operative phase. Accordingly, we have found that circulating and echocardiographic functional measurements reliably reflect cardiac damage. Based on our findings, we recommend defining objective criteria of hematological and biochemical indicators for confirming successful MI induction in experimental IR model 24 h post-IR using thresholds of cTnI > 12,000 ng/L, LDH > 780 U/L and AST > 960 U/L. Further, early-stage echocardiographic criteria at 72 h post-IR include functional parameters thresholds of FS ≤ 35%, FAC ≤ 55%, and EF ≤ 70%.

These proposed thresholds provide objective and reproducible criteria for the early identification of cardiac damage in the rat IR model, supporting their application in preclinical studies and enabling consistent comparisons across experiments. Importantly, to our knowledge, this study is the first to define ROC-derived objective thresholds for early *in vivo* validation of myocardial injury following IR in rats, providing a preliminary reference framework for confirming cardiac injury and informing future validation studies. By allowing reliable identification of injured animals shortly after surgery, without the need for terminal procedures, this approach offers a practical and efficient framework for early-stage model verification, reducing time and cost in preclinical research. Notably, the functional and histological similarity observed between sham-operated and non-injured animals suggests that the latter may serve as a useful comparator for selected functional or structural outcomes. However, further studies are required before considering non-injured animals as an alternative to sham-operated controls, particularly for molecular or inflammatory outcomes. Where appropriate, such an approach may contribute to reducing animal use, experimental costs, and study duration, in accordance with established ethical principles for animal research.

From a translational perspective, the integration of circulating and echocardiographic indicators closely reflects established clinical diagnostic pathways, thereby enhancing the relevance of preclinical IR models and strengthening their value for evaluating therapeutic interventions.

### Limitations

This study was conducted exclusively in young (6–9 weeks) male Sprague-Dawley rats. Therefore, physiological differences in females, aged animals, or other rodent strains may result in variable injury responses (Belkina et al., 2001). Male animals are commonly used in experimental cardiovascular research to minimize biological variability associated with hormonal fluctuations across the estrous cycle (Beery and Zucker, 2011). Similarly, Sprague-Dawley rats are widely used in preclinical MI models due to their well-characterized and reproducible responses to coronary artery ligation (Heusch et al., 2018).

The relatively small sample sizes of the sham-operated (n=9) and non-injured (n=10) groups represent an additional limitation and may reduce the precision and generalizability of the observed group comparisons and diagnostic estimates.

Furthermore, the retrospective classification used as the reference standard for the ROC analyses represents an inherent limitation. Although classification at Day 56 was based on multimodal functional and structural assessment, using these groups to evaluate earlier echocardiographic parameters may introduce selection bias and some degree of circularity, potentially contributing to inflation of the observed AUC estimates. In addition, echocardiographic and histological analyses were not performed blinded to group allocation, which may have introduced observer bias. Therefore, the ROC-derived thresholds should be considered internally derived and require validation in an independent cohort.

In addition, echocardiographic assessments were performed under ketamine/xylazine anesthesia, which may influence cardiac function and related echocardiographic measurements. Therefore, the proposed echocardiographic thresholds should be interpreted within the context of this anesthetic protocol and may require validation before application to studies using different anesthetic regimens.

Finally, assessments were restricted to the first week post-MI, and most measurements were obtained at a single time point. Given the dynamic kinetics of cardiac biomarkers and physiological responses following MI, broader temporal analyses would provide a more comprehensive characterization of injury progression. Nevertheless, the use of pre-defined and consistent points may facilitate methodological standardization across studies and improve the comparability and reliability of outcomes in other IR research protocols.

## Data Availability

The original contributions presented in the study are included in the article/supplementary material. Further inquiries can be directed to the corresponding authors.

## References

[B1] Babuin L. Jaffe A. S. (2005). Troponin: the biomarker of choice for the detection of cardiac injury. Cmaj 173, 1191–1202. doi: 10.1503/cmaj/051291 16275971 PMC1277047

[B2] Bachner-Hinenzon N. Ertracht O. Malka A. Leitman M. Vered Z. Binah O. . (2012). Layer-specific strain analysis: investigation of regional deformations in a rat model of acute versus chronic myocardial infarction. Am. J. Physiol. Heart Circ. Physiol. 303, H549–H558. doi: 10.1152/ajpheart.00294.2012 22777422

[B3] Beery A. K. Zucker I. (2011). Sex bias in neuroscience and biomedical research. Neurosci. Biobehav. Rev. 35, 565–572. doi: 10.1016/j.neubiorev.2010.07.002 20620164 PMC3008499

[B4] Belkina L. Saltykova V. Pshennikova M. (2001). Genetically determined differences in the resistance to myocardial infarction in Wistar and August rats. Bull. Exp. Biol. Med. 131, 529–532. doi: 10.1023/a:1012386115427 11586397

[B5] Bhindi R. Witting P. K. Mcmahon A. C. Khachigian L. M. Lowe H. C. (2006). Rat models of myocardial infarction. Thromb. Haemostasis 96, 602–610. doi: 10.1160/TH05-07-0514 17080217

[B6] Canty J. M. (2022). Myocardial injury, troponin release, and cardiomyocyte death in brief ischemia, failure, and ventricular remodeling. Am. J. Physiol. Heart Circ. Physiol. 323, H1–H15. doi: 10.1152/ajpheart.00093.2022 35559722 PMC9169835

[B7] Danese E. Montagnana M. (2016). An historical approach to the diagnostic biomarkers of acute coronary syndrome. Ann. Trans. Med. 4, 194. doi: 10.21037/atm.2016.05.19 27294090 PMC4885896

[B8] Daud E. Ertracht O. Bandel N. Moady G. Shehadeh M. Reuveni T. . (2021). The impact of empagliflozin on cardiac physiology and fibrosis early after myocardial infarction in non-diabetic rats. Cardiovasc. Diabetol. 20, 1–13. doi: 10.1186/s12933-021-01322-6 34215277 PMC8254247

[B9] De Villiers C. Riley P. R. (2020). Mouse models of myocardial infarction: comparing permanent ligation and ischaemia-reperfusion. Dis. Models Mech. 13, dmm046565. doi: 10.1242/dmm.046565 33361140 PMC7687859

[B10] Ertracht O. Liani E. Bachner‐Hinenzon N. Bar‐Am O. Frolov L. Ovcharenko E. . (2011). The cardioprotective efficacy of TVP1022 in a rat model of ischaemia/reperfusion. Br. J. Pharmacol. 163, 755–769. doi: 10.1111/j.1476-5381.2011.01274.x 21323905 PMC3111678

[B11] Ertracht O. Malka A. Atar S. Binah O. (2014). The mitochondria as a target for cardioprotection in acute myocardial ischemia. Pharmacol. Ther. 142, 33–40. doi: 10.1016/j.pharmthera.2013.11.003 24275322

[B12] Farag A. Mandour A. S. Hendawy H. Elhaieg A. Elfadadny A. Tanaka R. (2023). A review on experimental surgical models and anesthetic protocols of heart failure in rats. Frontiers in Veterinary Science 10, 1103229. doi: 10.3389/fvets.2023.1103229 PMC10083377 37051509

[B13] Frobert A. Valentin J. Magnin J.-L. Riedo E. Cook S. Giraud M.-N. (2015). Prognostic value of troponin I for infarct size to improve preclinical myocardial infarction small animal models. Front. Physiol. 6, 353. doi: 10.3389/fphys.2015.00353 26640441 PMC4661277

[B14] Heusch G. Bøtker H. E. Przyklenk K. Redington A. Yellon D. (2018). Practical guidelines for rigor and reproducibility in preclinical and clinical studies on cardioprotection. Basic Res. Cardiol. 113, 39. doi: 10.1007/s00395-018-0696-8 PMC6105267 30120595

[B15] Ibis M. A. Mencik N. O. Öztürk B. Karaburun M. C. Akpinar C. Akıncı A. . (2025). The relationship between sperm midpiece details and DNA fragmentation in human sperm. Reprod. Sci. 32, 2319–2330. doi: 10.1007/s43032-025-01887-7 40457018 PMC12270962

[B16] Jan M. F. Tajik A. J. (2017). Modern imaging techniques in cardiomyopathies. Circ. Res. 121, 874–891. doi: 10.1161/circresaha.117.309600 28912188

[B17] Jorge L. Rodrigues B. Rosa K. T. Malfitano C. Loureiro T. C. A. Medeiros A. . (2011). Cardiac and peripheral adjustments induced by early exercise training intervention were associated with autonomic improvement in infarcted rats: role in functional capacity and mortality. European Heart Journal 32 (7), 904–912. doi: 10.1093/eurheartj/ehq244 20675661

[B18] Kainuma S. Miyagawa S. Fukushima S. Tsuchimochi H. Sonobe T. Fujii Y. . (2017). Influence of coronary architecture on the variability in myocardial infarction induced by coronary ligation in rats. PLoS ONE 12 (8), e0183323. doi: 10.1371/journal.pone.0183323 PMC5570270 28837679

[B19] Li B. Yang W.-W. Yao B.-C. Chen Q.-L. Zhao L.-L. Song Y.-Q. . (2025). Liriodendrin alleviates myocardial ischemia-reperfusion injury via partially attenuating apoptosis, inflammation and mitochondria damage in rats. Int. J. Mol. Med. 55, 65. doi: 10.3892/ijmm.2025.5506 39981888 PMC11875722

[B20] Lindsey M. L. Brunt K. R. Kirk J. A. Kleinbongard P. Calvert J. W. de Castro Brás L. E. . (2021). Guidelines for in vivo mouse models of myocardial infarction. American Journal of Physiology-Heart and Circulatory Physiology 321 (6), H1056–H1073. doi: 10.1152/ajpheart.00459.2021 PMC8834230 34623181

[B21] Liu J. Rigel D. F. (2009). “ Echocardiographic examination in rats and mice,” in Cardiovascular Genomics: Methods and Protocols. (Totowa, NJ: Humana Press), 139–155. 10.1007/978-1-60761-247-6_8 19763926

[B22] Lucchesi B. R. (1990). Modulation of leukocyte-mediated myocardial reperfusion injury. Annu. Rev. Physiol. 52, 561–576. doi: 10.1146/annurev.physiol.52.1.561 2184767

[B23] Michael L. H. Ballantyne C. M. Zachariah J. P. Gould K. E. Pocius J. S. Taffet G. E. . (1999). Myocardial infarction and remodeling in mice: effect of reperfusion. Am. J. Physiol. Heart Circ. Physiol. 277, H660–H668. doi: 10.1152/ajpheart.1999.277.2.h660 10444492

[B24] Neckář J. Hye Khan M. A. Gross G. J. Cyprová M. Hrdlička J. Kvasilová A. . (2019). Epoxyeicosatrienoic acid analog EET-B attenuates post-myocardial infarction remodeling in spontaneously hypertensive rats. Clin. Sci. 133, 939–951. doi: 10.1042/CS20180728 PMC6492034 30979784

[B25] Nordbeck P. Bönhof L. Hiller K.-H. Voll S. Arias-Loza P. Seidlmayer L. . (2013). Impact of thoracic surgery on cardiac morphology and function in small animal models of heart disease: a cardiac MRI study in rats. PloS One 8, e68275. doi: 10.1371/journal.pone.0068275 23990872 PMC3749142

[B26] Pacher P. Nagayama T. Mukhopadhyay P. Bátkai S. Kass D. A. (2008). Measurement of cardiac function using pressure–volume conductance catheter technique in mice and rats. Nat. Protoc. 3, 1422–1434. doi: 10.1038/nprot.2008.138 18772869 PMC2597499

[B27] Pepe M. S. (2003). The Statistical Evaluation of Medical Tests for Classification and Prediction (Oxford, UK: Oxford University Press).

[B28] Roger V. L. (2013). Epidemiology of heart failure. Circ. Res. 113, 646–659. doi: 10.1161/circresaha.113.300268 23989710 PMC3806290

[B29] Song W. Tang Q. Teng L. Zhang M. Sha S. Li B. . (2023). Exercise for myocardial ischemia-reperfusion injury: a systematic review and meta-analysis based on preclinical studies. Microvasc. Res. 147, 104502. doi: 10.1016/j.mvr.2023.104502 36746363

[B30] Teichholz L. E. Kreulen T. Herman M. V. Gorlin R. (1976). Problems in echocardiographic volume determinations: echocardiographic-angiographic correlations in the presence or absence of asynergy. Am. J. Cardiol. 37, 7–11. doi: 10.1016/0002-9149(76)90491-4 1244736

[B31] Trueblood N. A. Inscore P. R. Brenner D. Lugassy D. Apstein C. S. Sawyer D. B. . (2005). Biphasic temporal pattern in exercise capacity after myocardial infarction in the rat: relationship to left ventricular remodeling. Am. J. Physiol. Heart Circ. Physiol. 288, H244–H249. doi: 10.1152/ajpheart.00042.2004 15358607

[B32] US National Research Council (2011). Guide for the Care and Use of Laboratory Animals: Eighth Edition. (Washington, DC: The National Academies Press). doi: 10.17226/12910

[B33] Virani S. S. Alonso A. Benjamin E. J. Bittencourt M. S. Callaway C. W. Carson A. P. . (2020). Heart disease and stroke statistics—2020 update: a report from the American Heart Association. Circulation 141, e139–e596. doi: 10.1161/cir.0000000000000757 31992061

[B34] Wever K. E. Hooijmans C. R. Riksen N. P. Sterenborg T. B. Sena E. S. Ritskes-Hoitinga M. . (2015). Determinants of the efficacy of cardiac ischemic preconditioning: a systematic review and meta-analysis of animal studies. PLoS ONE 10 (11), e0142021. doi: 10.1371/journal.pone.0142021 PMC4651366 26580958

[B35] Yang Y. Hao Y. Liu J. Yang N. Hu D. Sun Z. . (2022). Practice of reperfusion in patients with ST-segment elevation myocardial infarction in China: findings from the Improving Care for Cardiovascular Disease in China–Acute Coronary Syndrome project. Chin. Med. J. 135, 2821–2828. doi: 10.1097/cm9.0000000000002257 36728532 PMC9945072

[B36] Zacchigna S. Paldino A. Falcão-Pires I. Daskalopoulos E. P. Dal Ferro M. Vodret S. . (2021). Towards standardization of echocardiography for the evaluation of left ventricular function in adult rodents: a position paper of the ESC Working Group on Myocardial Function. Cardiovasc. Res. 117, 43–59. doi: 10.1093/cvr/cvaa110 32365197

[B37] Zheng Y. Zhang Q. Xu W. Dong Z. Huang L. Zhang L. . 2019 Wistar and Sprague-Dawlay rats demonstrate distinct features of metabolic profile after acute myocardial infarction.

[B38] Zuo Y. Yang S.-Y. Qian X.-K. Liu K. (2025). Analysis of hub genes temporal dynamics in cardiac ischemia/reperfusion injury: a bioinformatics and experimental study. Front. Physiol. 16, 1668586. doi: 10.3389/fphys.2025.1668586 41347019 PMC12672348

